# Driving pressure guided ventilation versus conventional lung protective strategy in morbid obese patients undergoing laparoscopic bariatric surgery: a prospective randomized controlled study

**DOI:** 10.1186/s12871-025-03431-1

**Published:** 2025-11-20

**Authors:** Mohamed Saed Elbehairy, Gehan Morsy Eid, Ashraf Elsayed Elzeftawy, Nabil Ali Elsheikh, Wail Ebrahim Messbah

**Affiliations:** Anesthesia, Surgical Intensive Care & Pain Management Department, Faculty of Medicine, Tanta University, Tanta, 31511 Egypt

**Keywords:** Driving pressure, Ventilation strategies, Individualized peep, Recruitment maneuver, Morbid obese

## Abstract

**Background:**

Mechanical ventilation in bariatric surgery presents unique challenges, requiring strategies that minimize intraoperative atelectasis, maintain adequate oxygenation, and lower the risk of postoperative pulmonary complications. The present study compared driving pressure–guided ventilation with conventional lung-protective ventilation in morbidly obese patients undergoing laparoscopic bariatric surgery.

**Methods:**

Sixty patients with a body mass index (BMI) of 40–50 kg/m², scheduled for laparoscopic bariatric surgery, were randomized according to intraoperative ventilation strategy into two groups: Group I (*n* = 30) received the conventional lung-protective strategy, and Group II (*n* = 30) received the driving pressure–guided ventilation strategy. After induction of pneumoperitoneum, a standardized lung recruitment maneuver was performed, after which ventilation strategies were applied according to group allocation: in Group I, positive end-expiratory pressure (PEEP) was maintained at 5 cmH₂O throughout surgery, whereas in Group II, PEEP was individualized to achieve the lowest driving pressure (DP).

**Results:**

The PaO₂/FiO₂ ratio showed significant improvement after the recruitment maneuver in both groups compared with baseline values. However, measurements obtained before the end of surgery and after extubation were significantly higher in Group II than in Group I (*P* < 0.001). Lung mechanics were also significantly better in Group II, with higher compliance, lower driving pressure, and reduced plateau pressure (Pplat). Intraoperative hypoxia requiring rescue therapy occurred in 10 patients (33.3%) in Group I compared with 2 patients (6.7%) in Group II, while postoperative hypoxia requiring supplementary oxygen was observed in 7 patients (23.3%) in Group I and in none of the patients in Group II.

**Conclusion:**

The adoption of driving pressure–based ventilation in laparoscopic bariatric surgery for morbidly obese patients was associated with improved oxygenation, optimized lung mechanics, and a lower risk of postoperative hypoxemia.

**Trial registration:**

The trial was registered prior to patient enrolment at ClinicalTrials.gov (NCT04861168, Date of registration: 27/4/2021).

## Background

 Obesity poses significant health risks, particularly affecting respiratory function and increasing complications during surgery. It has a negative impact on lung mechanics and function, which results in lower functional residual capacity, compliance, oxygenation, and increased atelectasis. Laparoscopic bariatric surgery has revolutionized the treatment of obesity by offering a minimally invasive alternative to traditional open surgery Compared with open surgery, it is associated with faster recovery, shorter hospital stays, and reduced postoperative discomfort [1].

Laparoscopic surgery is associated with more substantial perioperative respiratory and circulatory changes than open surgery. The process of abdominal insufflation itself results in known alterations to gas exchange and pulmonary mechanics [2].

Protective mechanical ventilation during anesthesia is designed to reduce lung damage and has been linked to a lower incidence of postoperative pulmonary complications (PPCs). A standard protective ventilation approach typically involves administering a low tidal volume (VT) along with a moderate, fixed level of PEEP [3, 4]. Nonetheless, utilizing low tidal volumes can reduce the functional capacity of the lungs, which may manifest as lung collapse [5]. Additionally, lung collapse can negatively affect ventilatory efficiency [6].

Alveolar recruitment maneuvers involve periodic hyperinflation of the lungs, aiding in the opening and sustaining of lung expansion in patients under anesthesia. Various techniques for implementing recruitment maneuvers have been shown to improve intraoperative lung mechanics and intraoperative oxygenation [7], unfortunately, this improvement is not sustained and needs either suitable PEEP to prevent lung re-collapse or periodic recruitment.

In their 2015 meta-analysis of ARDS patients, Amato et al. introduced driving pressure—the difference between plateau pressure and PEEP, according to the authors, driving pressure is a more accurate indicator of death than low VT and Pplat [8].

Driving pressure-guided ventilation during anesthesia enhances outcomes and reduces complications, particularly when the individualized PEEP is determined based on the driving pressure [9].

According to our knowledge, until now no research discusses the impact of DP guided ventilation in morbid obese patients under anesthesia. We suggested that utilizing a combination of recruitment maneuvers and individualized PEEP selection based on driving pressure would optimize intraoperative oxygenation and lung mechanics while ensuring patient safety and avoiding the harmful effects associated with excessive PEEP.

## Methods

This randomized, prospective, double-blind controlled trial enrolled 60 patients aged 18–60 years with a body mass index (BMI) of 40–50 kg/m² and classified as ASA physical status III, who were scheduled for laparoscopic bariatric surgery. Exclusion criteria included a recent history of severe respiratory disease, prior major lung surgery, or contraindications to the use of positive end-expiratory pressure (PEEP).

This study was approved by the Institutional Ethics Committee, Faculty of Medicine, Tanta University, Tanta, Egypt (NO: 34490/2/21) from August 2023 to April 2025. The study procedures follow the guidelines in the World Medical Association (WMA) Declaration of Helsinki. The trial was registered prior to patient enrolment at the ClinicalTrials.gov (NCT04861168, Date of registration: 27/4/2021). Patients who agreed to be part of our study submitted their informed consent.

### Randomization

Patients who met the designated criteria were randomly divided into two equal groups according to a mechanical ventilation strategy. This randomization process utilized computer-generated assignments, which were securely placed in sealed opaque envelopes to maintain confidentiality:

- Group I (*n* = 30) control group (Protective lung ventilation). Group II (*n* = 30) study group (Driving pressure-guided ventilation).

### Anesthesia management

After pre-oxygenation, general anesthesia was induced using propofol 2 mg/kg, fentanyl 1 mic/kg, and atracurium 0.5 mg/kg was given to facilitate endotracheal intubation. Neuromuscular blockade was achieved with bolus doses of atracurium 0.1 mg/kg and monitored using train-of-four (TOF) stimulation, targeting a deep block, and isoflurane at 1% minimum alveolar concentration were used to maintain anesthesia.

The Allen test was conducted, and the radial artery in the non-dominant hand was cannulated for invasive arterial blood pressure monitoring.

### Ventilation protocol

The DATEX-Ohmeda Avance CS2 anesthetic machine was used to ventilate the lungs in volume-controlled ventilation (VCV) mode. VT was set at 6–8 ml/kg of predicted body weight (PBW), PEEP of 5 cmH₂O, an inspiratory fraction of oxygen (FiO₂) of 0.5, and an inspiratory-to-expiratory ratio of 1:2, including a 10% end-inspiratory pause. The respiratory rate (RR) was adjusted to maintain end-tidal CO₂ (EtCO₂) between 35 and 45 mmHg. After anesthetic induction and hemodynamic stabilization, pneumoperitoneum was established and maintained with the insufflation of room-temperature CO₂ to achieve an intra-abdominal pressure of 12–15 mmHg.

 Five minutes after the establishment of pneumoperitoneum and assuring hemodynamic stability, recruitment was done by using a preset vital capacity on avance anesthesia machine that gave inflation pressure of 30 cmH_2_O for 30 seconds.

After recruitment, according to randomization conducted prior to surgery, ventilation strategy was determined based on PEEP as follows:

- Group I (control group): PEEP 5 cmH_2_O was set and maintained until the termination of surgery.
- Group II (study group): The least possible DP was achieved by gradually titrating PEEP in increments of 2 cmH_2_O. Each PEEP level was used for ten respiratory cycles, and DP was calculated during the last cycle using the formula: DP = plateau pressure – PEEP. If two PEEP levels resulted in the same DP, the lower PEEP level was selected and maintained until the termination of surgery.

### Rescue therapy

Lung recruitment was the first step in rescue intervention if desaturation (defined as spo2 < 95%) happened without severe hemodynamic impairment (hypotension or bradycardia) that interfere with gas exchange or oxygen delivery, ventilator dysfunction, or airway issues (endotracheal tube obstruction or kinking and bronchospasm). Fio_2_ was raised if the oxygen saturation did not improve [10].

Vasoactive drugs (atropine, ephedrine, and inotropes) were available for the management of hypotension and bradycardia. In the event of persistent hemodynamic instability, recruitment would be aborted and ventilation returned to a lower PEEP level. At surgery completion, FiO₂ was adjusted to 80%. Neuromuscular relaxation was reversed once monitoring demonstrated a train-of-four (TOF) ratio ≥ 0.9, confirming readiness for extubation [11].

Patients were extubated awake in a semi-sitting position after meeting satisfactory criteria and then transferred to the PACU, where they were positioned at approximately 45°. Oxygen was administered through a non-rebreathing facemask if saturation fell below 93% [12]. Supplemental oxygen continued until patients returned to their preoperative level of oxygen saturation.

### Measurements

Hemodynamic parameters, including heart rate (HR), mean arterial pressure (MAP), and SpO₂, were recorded at the following time points: preoperatively (T0), after induction of anesthesia (T1), following pneumoperitoneum insufflation (T2), 10 min after recruitment (T3), before the end of surgery (T4), and 30 min after surgery (T5). Lung mechanics—tidal volume (VT), peak airway pressure, plateau pressure (Pplat), PEEP, driving pressure (DP), and respiratory system compliance—were assessed at T1, T2, T3, T4, and T5. The PaO₂/FiO₂ ratio was calculated at T1, T3, T4, and T5.

The incidence of intraoperative hypoxemia (defined as SpO₂ < 95%), the need for rescue therapy, and postoperative pulmonary complications (PPCs), including hypoxemia (SpO₂ < 93%), requirement for supplemental oxygen, pulmonary infection, barotrauma, and respiratory failure, were also recorded.

The primary outcome was to assess the effect of driving pressure–guided ventilation compared with conventional lung-protective ventilation on intraoperative oxygenation in morbidly obese patients undergoing laparoscopic bariatric surgery. The secondary outcomes included the incidence of early PPCs, such as postoperative atelectasis and hypoxemia, the need for oxygen therapy, and the occurrence of barotrauma or respiratory failure.

### Statistical analysis and sample size calculation

A pilot study was conducted in a group of twenty adult patients (who were eventually eliminated from the final study) who had laparoscopic bariatric surgery. These patients were randomly divided into two groups, with an equal number of participants in each. The first group had general anesthesia with a traditional lung protection technique, while the second group received general anesthesia with driving pressure. The driving pressure group showed an increase in PaO2/FiO2 from 194.5_−_ ± 29.01 to 232 ± 42.4(*P* = 0.033). Therefore, A total of 25 patients were included in each group based on an α value of 0.05, a power of 95%, and the effect size was 1.04 as assessed by the G*Power statistical program version 3.1.9.7 (G-Power, Brunsbüttel, Germany). To accommodate for the possibility of dropouts, each group was increased to thirty patients.

### Statistical analysis of the data

Categorical data were summarized as numbers and percentages. For continuous data, normality was assessed using the Shapiro-Wilk test. Quantitative data were described using range (minimum and maximum), mean and standard deviation, Significance of the obtained results was judged at the 5% level.

The tests used were Chi-square test, Fisher’s Exact or Monte Carlo correction, Student t-test, and ANOVA with repeated measures.

## Results

**Fig. 1:**
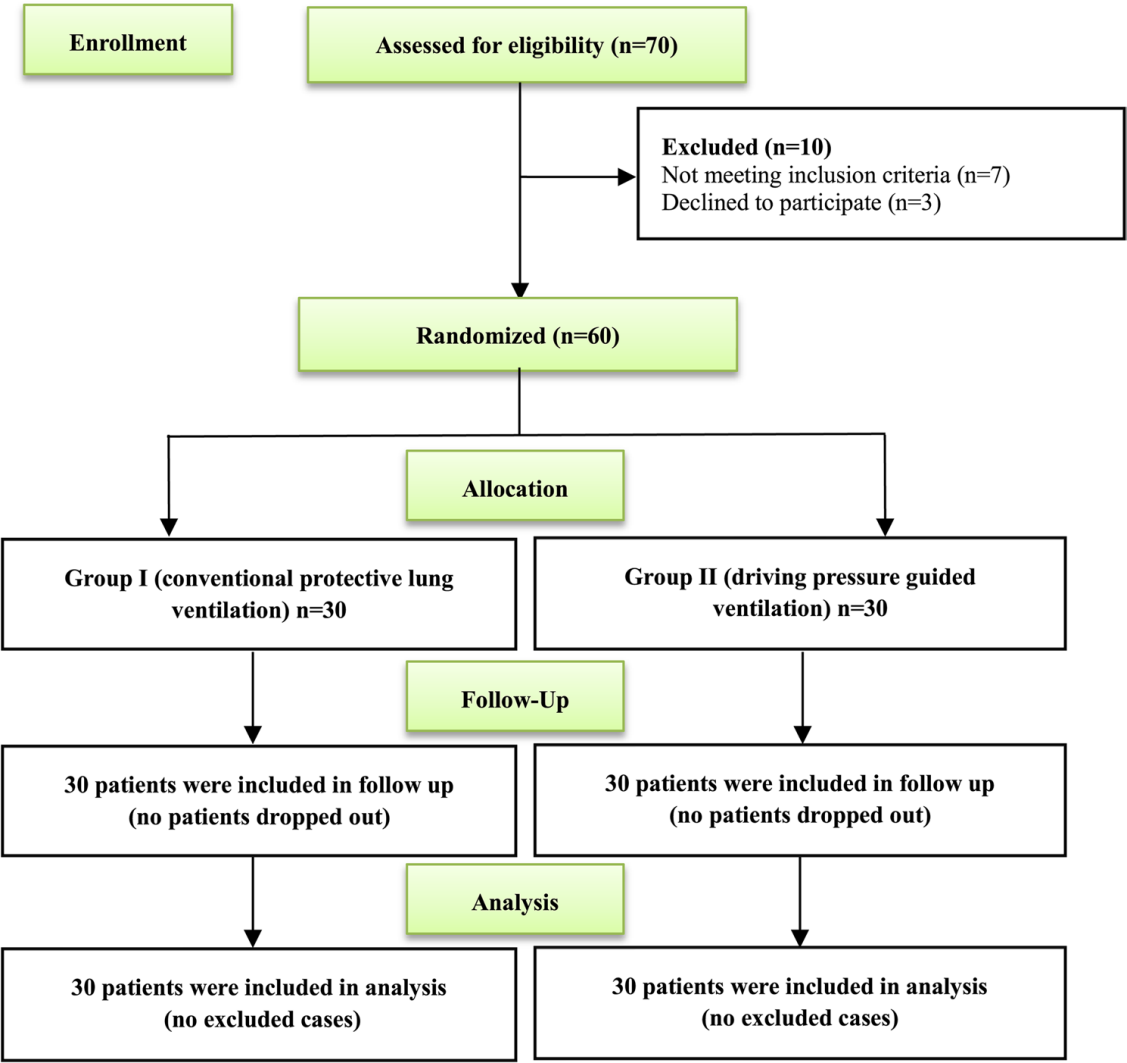
Consort flowchart of the study groups

There were in total 70 patients assessed for eligibility, and 10 patients were excluded; seven patients did not meet the inclusion criteria (five patients had a history of COPD, and two had a history of thoracic surgery) and three declined to be involved in the trial. The remaining 60 patients were randomly assigned to two groups (30 in each). All 60 patients were monitored, and their data were statistically examined (Fig. 1).

There was no discernible difference between the groups as regards patients characteristics including age, gender, height, BMI, and surgical data (Table 1).

**Table 1:** Comparison between the two studied groups as regard to patients’ characteristics and surgical data

	Group I (*n* = 30)		Group II (*n* = 30)		*p*	
	**No.**	**%**	**No.**	**%**		
Sex						
Male	9	30.0	10	33.3	0.781	
Female	21	70.0	20	66.7		
Age (years)						
Mean ± SD.	35.30 ± 8.79		36.57 ± 8.71		0.577	
Height (cm)						
Mean ± SD.	167.7 ± 5.42		166.8 ± 5.20		0.484	
Weight (kg)						
Mean ± SD.	128.1 ± 9.05		126.4 ± 9.58		0.491	
BMI (kg/m2)						
Mean ± SD.	45.58 ± 3.36		45.51 ± 3.58		0.936	

**Surgical data**					*P*
	**No.**	**%**	**No.**	**%**	
Type of operation					
Sleeve	23	76.7	22	73.3	0.766
Roux–en–Y / Bypass	7	23.3	8	26.7	
Duration of the operation (min.)	110.6 ± 19.58		108.8 ± 23.52		0.748
Length of pneumoperitoneum (min.)	101.2 ± 21.56		100.32 ± 17.53		0.863

**Fig. 2:**
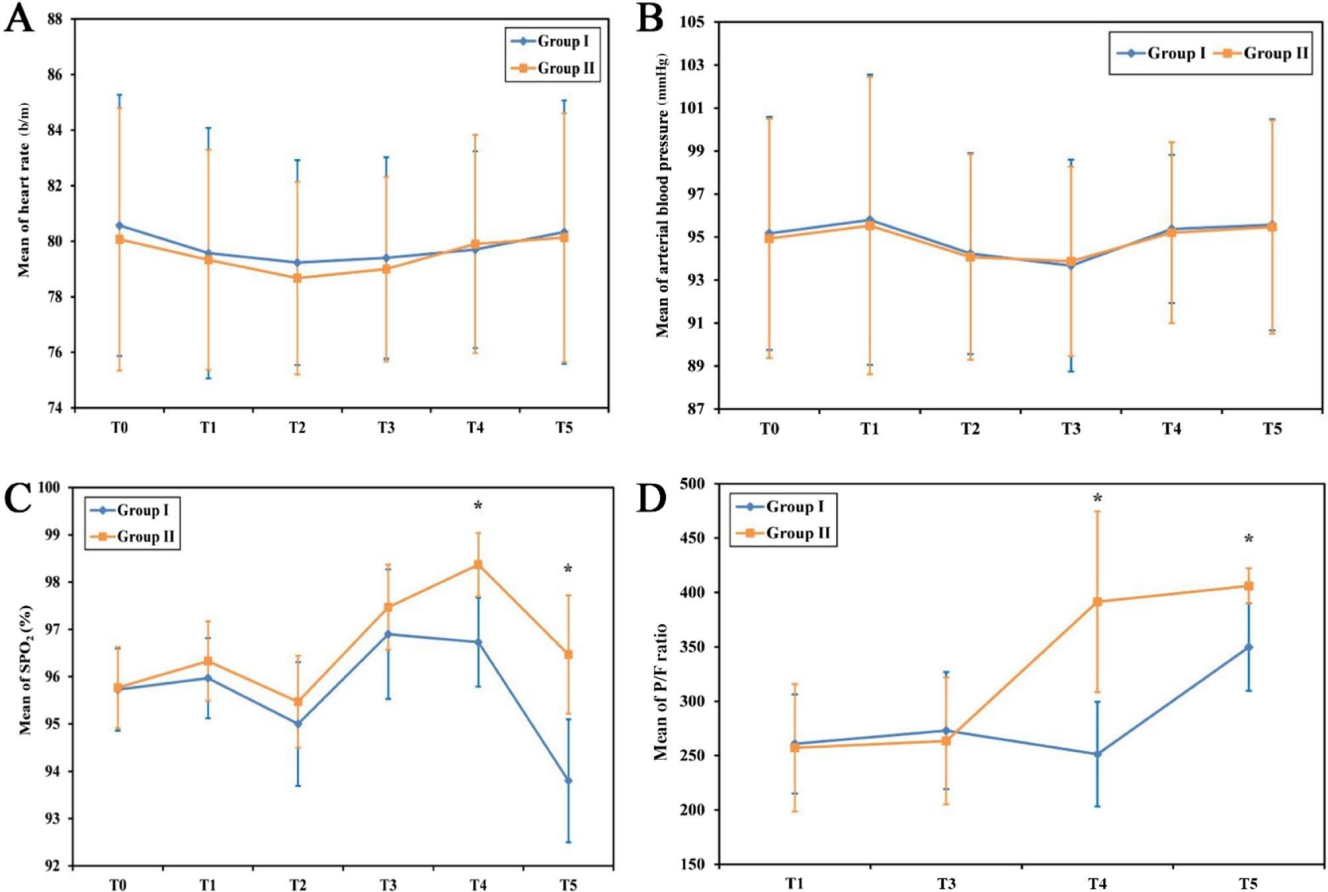
Comparison between the two studied groups as regard to haemodymics and oxygenation

Analysis of hemodynamic parameters (heart rate and invasive mean arterial pressure) from T0 to T5 demonstrated no significant differences between groups, nor within groups over time, while SpO₂ values were significantly higher in Group II compared to Group I at T4 and T5 (*P* < 0.001) (Fig. 2).

**Fig. 3:**
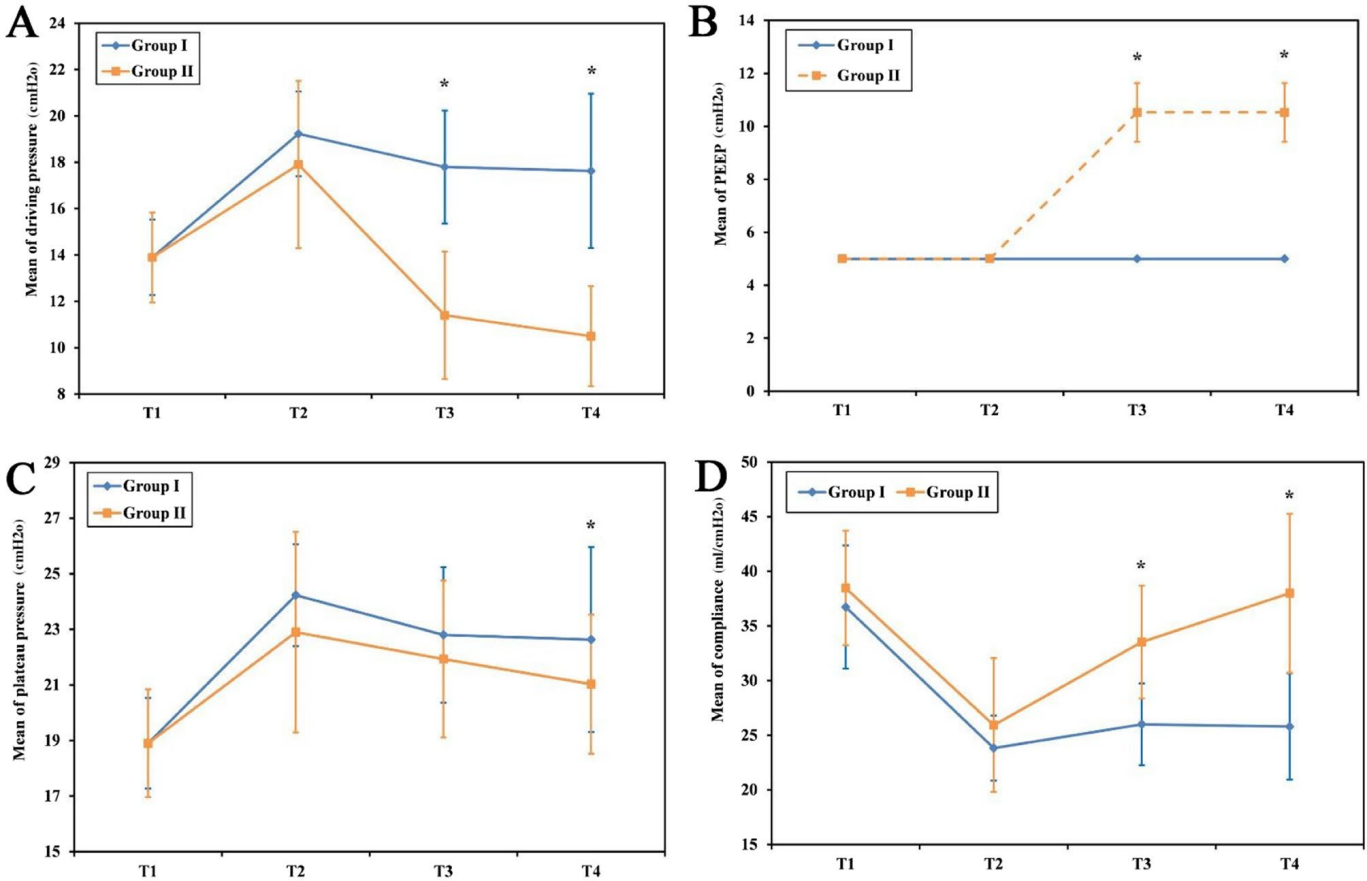
Comparison between the two studied groups as regard to lung mechanics

The PaO₂/FiO₂ ratio demonstrated significant improvement at T3 in both groups compared with baseline; however, at T4 and T5, the ratio was significantly higher in Group II than in Group I (*P* < 0.001) (Fig. 2D). Lung mechanics were significantly improved in Group II with higher lung compliance, lower DP, and lower Pplat while VT and peak pressure were similar in both groups (Fig. 3).

The mean PEEP values in Group II were 5 cmH₂O at T1 and T2, and 10.53 ± 1.11 cmH₂O at T3 and T4, whereas in Group I, PEEP was fixed at 5 cmH₂O throughout(Fig.3B). The mean values (± SD) for DP in Group I were 13.90 ± 1.63 cmH₂O at T1, 19.23 ± 1.83 cmH₂O at T2, 17.80 ± 2.44 cmH₂O at T3, and 17.63 ± 3.33 cmH₂O at T4. In Group II, DP values were 13.90 ± 1.94 cmH₂O at T1, 17.90 ± 3.61 cmH₂O at T2, 11.40 ± 2.75 cmH₂O at T3, and 10.50 ± 2.16 cmH₂O at T4 (Fig.3A).

**Fig. 4:**
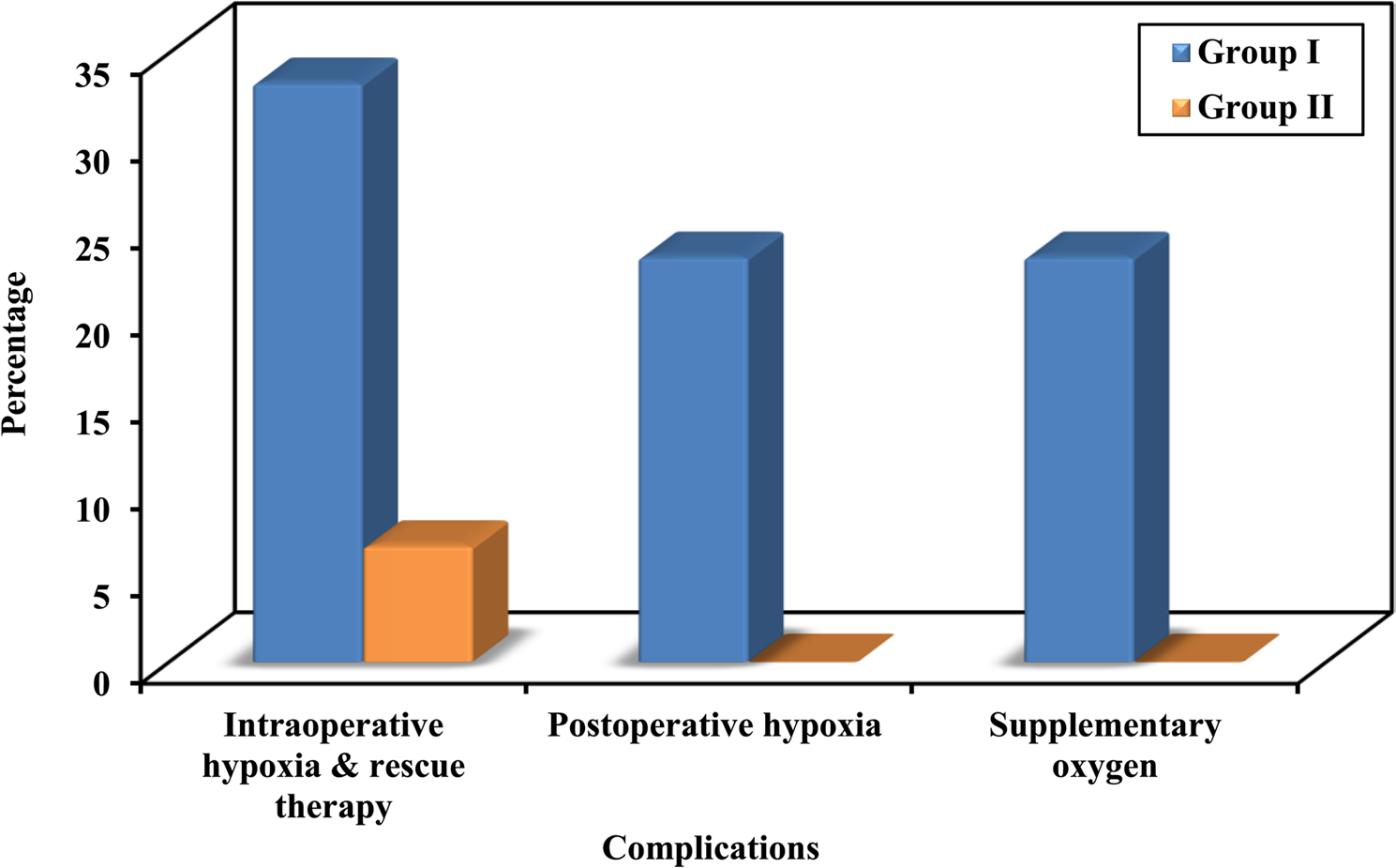
Comparison between the two studied groups as regard to intraoperative and postoperative complications

Regarding intraoperative and postoperative pulmonary complications, intraoperative hypoxia requiring rescue therapy occurred in 10 patients (33.3%) in Group I compared with 2 patients (6.7%) in Group II. Postoperative hypoxia requiring supplementary oxygen was observed in 7 patients (23.3%) in Group I, whereas no patients in Group II were affected (Fig.4). Intraoperative hypotension occurred in one patient (3%) in each group, and postoperative cough was reported in one patient (3%) in each group. No cases of barotrauma, respiratory failure, or pneumonia were observed in either group.

## Discussion

Laparoscopic bariatric surgery offers a minimally invasive alternative to open surgery, resulting in less tissue trauma, less pain, quicker recovery, and fewer complications. However, it is associated with notable alterations in pulmonary mechanics and gas exchange due to abdominal insufflation.

Using low VT and PEEP is part of protective mechanical ventilation during anesthesia, which attempts to minimize lung injury and has been linked to a decrease in PPCs [3, 4]. On the other hand, low VT may cause the lung’s functional volume to decrease, which could lead to lung collapse, and impairment in ventilatory efficient [6].

Alveolar recruitment maneuvers involve periodic hyperinflation of the lungs, aiding in the opening and sustaining of lung expansion in patients under anesthesia. Various techniques for implementing recruitment maneuvers have been shown to improve intraoperative lung mechanics and oxygenation during general anesthesia [7]. Unfortunately, this improvement is not sustained and needs either suitable PEEP to prevent lung re-collapse or periodic recruitment.

In their 2015 meta-analysis study for ARDS patients, Amato et al. suggested that driving pressure is a more reliable indicator of death than low VT and Pplat [8].

“Baby lung” concept, or functional lung size, refers to the volume of healthy lungs that can be ventilated, which highlights that ARDS patients possess a lung volume comparable to that of a 5–6-year-old child. This concept emphasizes that the respiratory system compliance is directly linked to the dimensions of the “baby lungs”; thus, in ARDS lungs it is important to adjust ventilation based on the VT/baby lung ratio rather than the VT/kg ratio, as smaller baby lungs increase the risk of ventilator-induced lung injury (VILI). Conditions like atelectasis and consolidation can further reduce functional lung size, leading to decreased compliance and clinically significant hypoxemia. Over-distension or under-ventilation beyond this functional size raises driving pressure, which is minimized when PEEP maintains alveoli at functional residual capacity. Consequently, driving pressure serves as a practical guide for individualized ventilation strategies based on each patient’s functional lung size, being easier to calculate than static compliance, since driving pressure is derived from two simple pressures [Pplat– PEEP]. In contrast, static compliance involves a more complex calculation that incorporates both pressures and tidal volumes [VT/(Pplat– PEEP)], making it more challenging to compute [13, 14].

Driving pressure consists of transpulmonary and cross-chest wall components. In the absence of spontaneous breathing, changes in driving pressure primarily reflect changes in transpulmonary pressure, which correlates linearly with lung stress and strain. These factors are significant contributors to VILI [15, 16].

This study hypothesized that integrating recruitment maneuvers and individualized PEEP selection based on driving pressure would optimize intraoperative oxygenation and lung mechanics while ensuring patient safety and avoiding the harmful effects associated with excessive PEEP.

In the present study, we found that the lung recruitment maneuver improved intraoperative oxygenation in both groups compared with baseline. However, this improvement-reflected by a reduced incidence of intraoperative hypoxia and a lower need for rescue therapy-was sustained only when individualized PEEP was applied by gradually titrating PEEP in 2 cmH₂O increments to achieve the lowest possible driving pressure in Group II. In contrast, in Group I, repeated recruitment maneuvers were required to prevent intraoperative hypoxia.Supporting these findings, Zhang et al. demonstrated that individualized PEEP based on minimum driving pressure decreased atelectasis severity and improved oxygenation in abdominal surgery patients. Similarly, Nestler et al. showed that in obese patients undergoing laparoscopic surgery, combining RM with individualized PEEP improved lung volume and oxygenation during anesthesia, though these benefits did not last post-extubation [17].

Lung recruitment through sustained inflation at a preset vital capacity of 30 cmH_2_O for 30 s proved to be both effective and safe, with no significant changes in hemodynamics or pulmonary complications. These findings align with the results of Severac et al. [10], who conducted a randomized trial comparing standard protective ventilation with a recruitment group that received protective ventilation along with systematic recruitment maneuvers. In their study, the recruitment maneuvers-maintained airway pressure at 30 cmH_2_O for 30 s every 30 min. They reported that this approach was well-tolerated, with no significant instances of bradycardia or hypotension. Furthermore, patients in the control group required rescue recruitment maneuvers due to elevated plateau pressures or intraoperative hypoxemia.

In our study on lung mechanics, we found that driving pressure-guided ventilation targeting the lowest possible driving pressure significantly improved intraoperative lung mechanics. This was evidenced by increased respiratory system compliance, decreased lung atelectasis, and lower plateau and driving pressures in the study group Our results are consistent with those of Park et al. [18], who showed that driving pressure–guided ventilation enhanced pulmonary mechanics during surgery and reduced the requirement for rescue ventilation. Likewise, Yang et al. reported that a driving pressure–guided strategy lowered intraoperative driving pressure and increased respiratory compliance, although the overall incidence of PPCs was similar between the two groups [9].

It is evident that reducing intraoperative driving pressure and enhancing intraoperative oxygenation and lung mechanics in the study group led to a decreased incidence of postoperative hypoxemia, thereby reducing the need for supplementary oxygen in the DP group. This is consistent with a meta-analysis by Neto et al. [19], which revealed that the probability of PPCs increased by 1.16 for every 1 cmH_2_O rise in driving pressure, highlighting the risks associated with high PEEP when it led to increased driving pressure. Conversely, high PEEP could reduce the risk of complications if it resulted in decreased driving pressure.

Various radiological modalities can be used to assess intraoperative atelectasis and evaluate the effectiveness of ventilation strategies to counteract it; however, their cost and limited availability restrict routine use in the operating room. Based on the results of this study, we recommend driving pressure–guided ventilation in bariatric surgeries as a cost-effective, accessible, and reliable method to ensure safe and effective mechanical ventilation.

This study is limited by its single-center design and relatively small sample size, although the findings were statistically significant. Moreover, because major postoperative pulmonary complications are uncommon in our institution, except for atelectasis and hypoxemia, the effect of driving pressure–guided ventilation on other PPCs could not be fully assessed.

## Conclusion

Driving pressure guided ventilation resulted in improvement of intraoperative oxygenation, lung mechanics and decrease postoperative hypoxemia in morbidly obese patients undergoing laparoscopic bariatric surgery.

## Data Availability

Data is available on reasonable requests from the corresponding author.
